# The effect of histological and subclinical chorioamnionitis and funisitis on breathing effort in premature infants at birth: a retrospective cohort study

**DOI:** 10.1007/s00431-024-05815-w

**Published:** 2024-10-25

**Authors:** Timothy J. R. Panneflek, Janneke Dekker, Kristel L. A. M. Kuypers, Lotte E. van der Meeren, Graeme R. Polglase, Stuart B. Hooper, Thomas van den Akker, Arjan B. te Pas

**Affiliations:** 1 Division of Neonatology, Department of Paediatrics, Willem-Alexander Children’s Hospital, Leiden University Medical Centre, P.O. Box 9600, 2300 RC Leiden, The Netherlands; 2 Department of Pathology, Leiden University Medical Centre, Leiden, The Netherlands; 3 Department of Pathology, Erasmus Medical Centre, Rotterdam, The Netherlands; 4 The Ritchie Centre, Hudson Institute of Medical Research, Melbourne, VIC Australia; 5 Department of Obstetrics and Gynaecology, Monash University, Melbourne, VIC Australia; 6 Department of Obstetrics, Leiden University Medical Centre, Leiden, The Netherlands; 7 Athena Institute, VU University, Amsterdam, The Netherlands

**Keywords:** Chorioamnionitis, Funisitis, Breathing, Resuscitation, Neonatology

## Abstract

Antenatal inflammation in the form of chorioamnionitis (fetal membranes; HCA) and funisitis (umbilical vessels; FUN) is a major risk factor for preterm birth. Exposure to HCA + FUN affects infants by releasing mediators that may suppress respiratory drive. While the association between clinical chorioamnionitis (CCA) and (depressed) spontaneous breathing has been described, we have investigated the association between breathing and HCA + FUN. Infants born < 30 weeks’ gestation with available placental pathology assessments were included. Infants were compared at multiple levels: infants with vs without HCA + FUN (comparison 1) and infants with subclinical HCA + FUN vs infants without any chorioamnionitis (comparison 2). The primary outcome was breathing effort, defined as minute volume (MV) of spontaneous breathing in the first 5 min after birth. We also assessed tidal volume (Vt), respiratory rate (RR), heart rate (HR), oxygen saturation (SpO_2_) and oxygen requirement (FiO_2_). Regression analyses were performed to control for confounding factors. One hundred eighty-six infants were included (*n* = 75 infants with HCA + FUN vs. *n* = 111 infants without HCA + FUN). Comparison 1: Infants with HCA + FUN had lower gestational ages 26^+5^ (25^+0^–28^+1^; median (IQR) and lower birthweights (mean ± SD; 943 ± 264) compared to infants without HCA + FUN (28^+4^ (27^+0^–29^+1^) weeks, *p* < 0.001 and 1023 ± 270 g, *p* = 0.049). Comparison 2: Subclinical HCA + FUN was diagnosed in 46/75 HCA + FUN infants. Infants with subclinical HCA + FUN had lower gestational ages (26^+6^ (25^+1^–28^+3^) vs. 28^+4^ (27^+2^–29^+1^) weeks, *p* < 0.001) without significant differences for birthweights (987 ± 248 vs. 1027 ± 267 g, *p* = 0.389) compared to infants without any chorioamnionitis (*n* = 102 infants). After adjustment, HCA + FUN was associated with lower MV (*p* = 0.025), but subclinical HCA + FUN was not (*p* = 0.226). HCA + FUN and subclinical HCA + FUN were associated with lower Vt (*p* = 0.003; *p* = 0.014), SpO_2_ at 5 min (*p* = 0.021; 0.036) and SpO_2_/FiO_2_ ratio (*p* = 0.028; *p* = 0.040).

*Conclusion*: HCA + FUN and subclinical HCA + FUN are associated with reduced oxygenation and parameters that reflect breathing effort in premature infants at birth.

**Table:** 

**What is Known:**
• *Acute antenatal inflammation, in the form of chorioamnionitis (fetal membranes) and funisitis (umbilical vessels), affects a large proportion of premature infants.* • *Clinical chorioamnionitis is associated with reduced breathing effort and oxygenation in premature infants at birth.*
**What is New:**
• *Histological and subclinical chorioamnionitis and funisitis are associated with reduced breathing effort parameters and oxygenation in premature infants at birth.*

**Supplementary Information:**

The online version contains supplementary material available at 10.1007/s00431-024-05815-w.

## Introduction

As premature infants often struggle to aerate their lungs at birth due to their immature respiratory system, they often require respiratory support to establish effective gas exchange [1]. Since respiratory support is initially provided non-invasively (via continuous positive airway pressure (CPAP) or intermittent positive pressure ventilation (iPPV)), laryngeal patency is key to ensure that those pressures are applied to the lungs [1]. Experimental and clinical studies have shown that, at birth, the larynx is mostly closed and only opens during spontaneous breathing, until a stable breathing pattern is established [2, 3]. Therefore, stimulating and supporting spontaneous breathing is essential to ensure that non-invasive respiratory support is effective.

Spontaneous breathing is affected by multiple factors such as arousal state, oxygenation level and inflammation [1]. Inflammation potentially leads to the release of mediators, such as prostaglandins and adenosine, that can inhibit the respiratory drive generated within the brainstem [4]. These mediators are also released during hypoxia, although the inhibitory effect of hypoxia is thought to be mediated by chemoreceptors that signal via the upper lateral pons directly into the brainstem [4, 5]. As inflammation is also known to induce hypoxia and vice versa, a potential negative feedback loop depressing spontaneous breathing could affect premature infants subjected to inflammation [4, 6, 7].

A large proportion of premature infants are subjected to antenatal inflammation of the fetal membranes (chorioamnionitis) and umbilical vessels (funisitis), which is defined according to clinical (CCA) and histological criteria (histological chorioamnionitis (HCA) and funisitis (FUN)) [8]. CCA is a clinical infection diagnosed by the suspected Triple I criteria that identify intrauterine infection and inflammation, the current standard for the diagnosis of CCA [9]. HCA + FUN is defined as acute inflammation in the membranes with maternal neutrophils infiltrating in either subchorionic fibrin, chorion, amnion, decidua, umbilical vessels and/or Wharton’s jelly [10]. CCA and HCA + FUN are associated entities, but not mutually exclusive: HCA + FUN often occurs without CCA (subclinical HCA + FUN) and CCA can sometimes occur without HCA + FUN (clinical infection without evident placental origin) [11]. As chorioamnionitis (clinical, histological and subclinical) and funisitis are associated with an increased requirement for respiratory support at birth, an inflammatory-mediated respiratory depression may influence the spontaneous breathing of infants affected by antenatal inflammation [12–16].

Following on from our previous finding that CCA is associated with reduced spontaneous breathing at birth [16], in this study, we investigated two relationships in premature infants at birth: (i) the relationship between histopathological evidence of antenatal inflammation (i.e. HCA + FUN) and spontaneous breathing, and (ii) the relationship between histopathological evidence of antenatal inflammation in the absence of maternal clinical symptoms (i.e. subclinical HCA + FUN) on spontaneous breathing.

## Methods

Methods have been previously described in a case–control study matching premature infants with CCA to controls without CCA [16]. In brief, a retrospective cohort study was performed on infants born in the Leiden University Medical Centre (LUMC) at < 30 weeks’ gestation between January 2016 and April 2021. Data were collected from respiratory function monitors (RFMs; Advanced Life Diagnostics resuscitation monitor, Weener, Germany) and electronic health records.

**Fig. 1:**
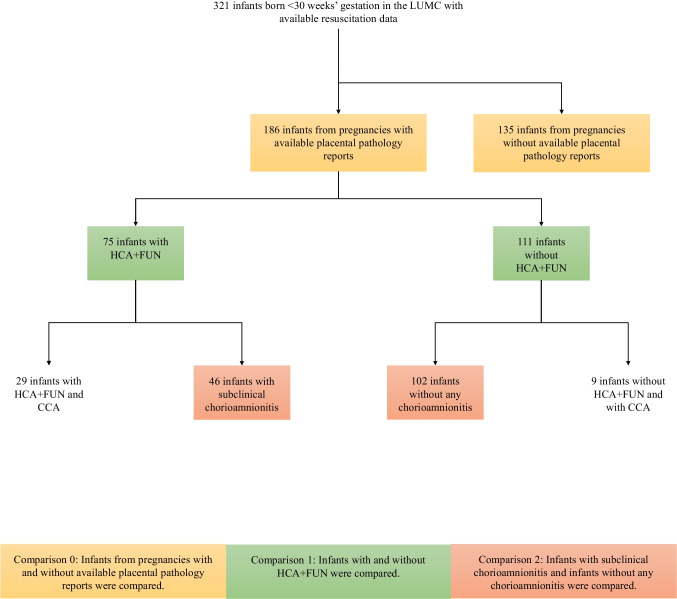
Flowchart diagram. Flowchart of comparisons between different groups

Infants were included in the study when RFM data was available and they were compared at three different levels (Fig. 1). Comparison 0: infants from pregnancies with available placental pathology reports were compared to infants from pregnancies without available placental pathology reports. Comparison 1: infants with histological chorioamnionitis and/or funisitis (HCA + FUN) were compared to infants without HCA + FUN. Comparison 2: infants with subclinical HCA + FUN were compared to infants without any chorioamnionitis (i.e. without HCA + FUN or CCA) (Fig. 1). In the LUMC, placental pathology is ordered by the attending obstetrician upon indications listed in Supplementary file 1 when it might contribute to obstetrical evaluation of the pregnant woman. HCA + FUN was defined according to the placental clinical pathology report where anatomical localisation of inflammation was recorded (i.e. chorion, amnion and umbilical vessels) without grading or staging the inflammation.

Respiratory support in the delivery room was provided using a Neopuff™ infant T-piece resuscitator with a facemask (Fisher & Paykel Healthcare, Auckland, New Zealand) in combination with a visible RFM. Respiratory support started with CPAP 5–8 cm H_2_O, but in case of apnoea and/or bradycardia, initial inflations of 3–15 s or iPPV were administered with a frequency of 40–60 inflations per minute, a positive end-expiratory pressure of 5–8 cm H_2_O and a peak inspiratory pressure (PIP) of 20–25 cm H_2_O. Fraction of inspired oxygen (FiO_2_) was commenced at 0.3 and titrated based on the 25th percentile of a published normogram [17]. Collected RFM data consisted of physiological measurements in the first 5 min after birth using a Masimo SET pulse oximeter probe (Masimo Radical, Masimo Corporation, Irvine, California, USA), a portable oxygen analyser (AX300-I Teledyne Analytical Instruments, CA, USA) and a variable orifice flow sensor (Avea Varflex Flow Transducer, Carefusion, Yorba Linda, CA, USA) recorded digitally using the Polybench physiological software (Applied Biosignals, Weener, Germany). Pulmochart software (Applied Biosignals) was used to calculate respiratory outcomes corrected for birth weight. All physiological data was assessed blinded to the groups.

The primary outcome was measurable breathing effort in the first 5 min after birth, measured as minute volume of spontaneous breathing. Minute volume was calculated as the product of the average inspiratory tidal volume on CPAP with mask leak < 75% and respiratory rate per minute of breathing independent of respiratory support provided during this time period. The primary outcome was calculated per minute. Additional physiological parameters assessed in the first 5 min after birth consisted of average: inspiratory drive (tidal volume corrected by inspiratory time; a reflection of depth of breathing [18]), incidence and duration of apnoea, cessation of breathing > 10 s, inter-breath interval coefficient of variation (COV, a higher COV indicates a less stable breathing pattern [19]), CPAP and PIP levels, heart rate (HR), oxygen saturation (SpO_2_) and FiO_2_. Infants were considered stable according to the following criteria: HR ≥ 100, SpO_2_ ≥ 85%, FiO_2_ ≤ 40% during spontaneous breathing. Data on respiratory support in the delivery room and NICU were also collected (i.e. initial inflations, caffeine, iPPV, surfactant administration and intubation rate).

Demographics comprised of maternal characteristics: maternal age, parity, multiple pregnancy, mode of birth, type of anaesthesia, antenatal corticosteroids, maternal intrapartum antibiotics,^1^ CCA^2^ and anatomical location of inflammation, and neonatal characteristics: gestational age, birth weight, sex, Apgar scores and umbilical pH.

### Statistical analysis

Statistical analyses were performed with IBM SPSS Statistics V.29.0 (IBM Software, Chicago, Illinois, USA, 2022) and R version 4.4.0 (R: The R Project for Statistical Computing (r-project.org)) within Studio version 4.4.0 (R Studio, Boston, MA, USA, 2024). In comparison 0, baseline characteristics (i.e. demographic data) were compared between infants with vs infants without available placental pathology (Fig. 1 and Supplemental file 2). Baseline characteristics were also compared between infants with and without HCA + FUN (comparison 1) and between infants with subclinical HCA + FUN and infants without any chorioamnionitis (comparison 2) (Fig. 1).

Respiratory, additional physiological and respiratory support parameters were compared between infants with and without HCA + FUN (comparison 1) and between infants with subclinical HCA + FUN and infants without any chorioamnionitis (comparison 2) after adjusting for potential confounding factors described below (Fig. 1).

Categorical data were described as *n* (%) and analysed with a Pearson chi^2^-test or Fisher’s exact test. Normality for continuous data was assessed via inspection of histograms and using the Shapiro-Wilkinson test. Parametric data was presented as mean ± standard deviation and analysed with an Independent samples *T*-test, while non-parametric data was presented as median (interquartile range) and analysed with a Mann–Whitney *U* test. Outcomes for “time until a physiological event^3^” were presented as median (interquartile range) and analysed with a Log-rank test of the Kaplan–Meier survival curve. Missing data is noted in the corresponding table.

Taking into account that gestational age, birthweight, antenatal corticosteroids, mode of delivery and general anaesthesia could affect spontaneous breathing at birth, multiple regression analyses were performed to adjust for these covariates when baseline differences between groups in these variables were significant [16, 20–22]. Birthweight was exempted as a covariate, if gestational age was also different between groups, due to the likely collinearity between birthweight and gestational. Likewise, the mode of delivery was exempted if general anaesthesia was significant. Raw data of respiratory and additional physiological outcomes are presented and compared for comparisons 1 and 2 in Supplementary file 3.

The primary outcome, measurable breathing effort, was compared over time in comparisons 1 and 2 using a linear mixed-effects model accounting for multiple measurements in the same infant. Fixed effects in the regression analyses consisted of (subclinical) HCA + FUN, time, (subclinical) HCA + FUN*time and the other aforementioned covariates. An unstructured variance–covariance matrix was used. The outcome of the linear mixed-effects model was described as estimated marginal mean ± standard error for the (subclinical) HCA + FUN fixed effect. The *p*-value for the (subclinical) HCA + FUN covariate assesses the difference in average breathing effort between groups. The p-value for the (subclinical) HCA + FUN*time fixed effect informs on differences in breathing effort between groups over time in the first 5 min after birth.

All other outcomes were compared with generalised linear models, binary logistic regression or Cox regression and presented as estimated marginal means ± standard error, odds ratios (95% confidence interval; 95% CI) and median (interquartile range), respectively, with adjusted *p*-values. For generalised linear models, only adjusted *p*-values are displayed in the results section.

Post hoc analysis was employed for inspiratory tidal volume, respiratory rate, inspiratory drive and inter-breath variability to analyse temporal changes.

A two-sided *p*-value < 0.05 was considered significant.

### Results

Resuscitation data were available for 321 infants born < 30 weeks’ gestation in the LUMC between 2016 and 2021; 186 placentas were examined for pathology of which 75 (40%) placentas had evidence of HCA + FUN and 111 (60%) did not (Fig. 1).

### Baseline Characteristics

#### Comparison 1

Infants with HCA + FUN had lower gestational ages (infants with HCA + FUN vs. infants without HCA + FUN; 26^+5^ (25^+0^–28^+1^) vs. 28^+4^ (27^+0^–29^+1^) weeks, *p* < 0.001), lower birthweights (943 ± 264 vs. 1023 ± 270, *p* = 0.049) and lower Apgar scores at 1 min (5 (2–7) vs. 6 (3–7), *p* = 0.006) compared to infants without HCA + FUN (Table 1). Infants with HCA + FUN had a lower incidence of caesarean deliveries (13 (28%) vs. 70 (69%), *p* < 0.001) and general anaesthesia (10 (13%) vs. 28 (25%), *p* = 0.048), while they had higher exposure to maternal intrapartum antibiotics (31 (41%) vs. 23 (21%), *p* = 0.002) and antenatal corticosteroids (55 (73%) vs. 64 (58%), *p* = 0.043) (Table 1).

#### Comparison 2

Infants with subclinical HCA + FUN (*n* = 46) had lower gestational ages (infants with subclinical HCA + FUN vs. infants without any chorioamnionitis; 26^+6^ (25^+1^–28^+3^) vs. 28^+4^ (27^+2^–29^+1^) weeks, *p* < 0.001) were less often born after caesarean Sect. (13 (28%) vs. 70 (69%), *p* < 0.001) and had lower Apgar scores at 1 min (3 (2–6) vs. 5 (4–7), *p* = 0.005) compared to infants without any chorioamnionitis (*n* = 102) (Table 1).

**Table 1:** Baseline characteristics of infants with and without chorioamnionitis and funisitis

	Infants with HCA + FUN (*n* = 75)	Infants without HCA + FUN (*n* = 111)	*p*-value
**Baseline characteristics**			
Maternal age (yrs)	31 ± 5	31 ± 5	0.394^a^
Primiparity	47 (63%)	65 (59%)	0.574^b^
Multiple gestation	21 (28%)	34 (31%)	0.700^b^
Caesarean delivery	22 (29%)	76 (69%)	< 0.001^b^
General anaesthesia	10 (13%)	28 (25%)	0.048^b^
Full course of antenatal corticosteroids	55 (73%)	64 (58%)	0.043^b^
Maternal intrapartum antibiotics	31 (41%)	23 (21%)	0.002^b^
Clinical chorioamnionitis	29 (39%)	9 (8%)	< 0.001^b^
Anatomical location of inflammation			
Chorionitis	16 (21%)		
Chorioamnionitis	18 (24%)		
Funisitis	41 (55%)		
Gestational age (weeks)	26^+5^ (25^+0^–28^+1^)	28^+4^ (27^+0^–29^+1^)	< 0.001^c^
Birthweight (g)	943 ± 264	1023 ± 270	0.049^b^
Small for gestational age	8 (11%)	44 (40%)	< 0.001^b^
Male	32 (43%)	54 (49%)	0.422^b^
Apgar score 1 min	4 (2–6)	5 (3–7)	0.006^c^
Apgar score 5 min	7 (6–8)	8 (6–9)	0.097^c^
Umbilical pH	7.26 ± 0.10	7.25 ± 0.11	0.772^a^
	Infants with subclinical HCA + FUN (*n* = 46)	Infants without any chorioamnionitis (*n* = 102)	
Maternal age (yrs)	31 ± 5	31 ± 5	0.837^a^
Primiparity	28 (61%)	60 (59%)	0.814^b^
Multiple gestation	8 (17%)	28 (28%)	0.187^b^
Caesarean delivery	13 (28%)	70 (69%)	< 0.001^b^
General anaesthesia	9 (20%)	22 (22%)	0.782^b^
Full course of antenatal corticosteroids	32 (70%)	61 (60%)	0.140^b^
Maternal intrapartum antibiotics	10 (22%)	15 (15%)	0.291^b^
Anatomical location of inflammation			
Chorionitis	15 (33%)		
Chorioamnionitis	9 (20%)		
Funisitis	23 (50%)		
Gestational age (weeks)	26^+6^ (25^+1^–28^+3^)	28^+4^ (27^+2^–29^+1^)	< 0.001^c^
Birthweight (g)	987 ± 248	1027 ± 267	0.389^b^
Small for gestational age	0 (0%)	44 (40%)	0.057^d^
Male	24 (52%)	49 (48%)	0.641^b^
Apgar score 1 min	3 (2–6)	5 (4–7)	0.005^c^
Apgar score 5 min	7 (6–8)	8 (6–9)	0.028^c^
Umbilical pH	7.3 ± 0.1	7.3 ± 0.1	0.683^a^

Umbilical pH represents the umbilical artery or vein pH

Small for gestational age is defined as infants with birthweight < p10 for gestational age

^a^Independent samples *T*-test

^b^Chi^2^-test

^c^Mann-Whitney *U* test

^d^Fisher’s exact test

Umbilical pH data missing for 18/75 (24%), 31/111 (28%), 9/46 (20%) and 28/102 (27%) infants

### Respiratory parameters in the first 5 min after birth

#### Comparison 1

After adjustment for the covariates (see above), HCA + FUN was associated with lower minute volume (38 ± 20 vs. 74 ± 18 mL/kg/min, *p* = 0.029) without a significant change in minute volume over time (*p* = 0.496). HCA + FUN was associated with lower tidal volume (*p* = 0.006), lower inspiratory drive (*p* < 0.001) and a greater variability (assessed as COV) of the inter-breath interval (*p* = 0.019), while the respiratory rate was not significantly affected by the presence of HCA + FUN (Table 2).

#### Comparison 2

After adjustment, subclinical HCA + FUN was not associated with lower minute volume (64 ± 17 vs. 91 ± 12 mL/kg/min, *p* = 0.220) nor with a significant change in minute volume over time (*p* = 0.541). Subclinical HCA + FUN was also associated with lower tidal volume (*p* = 0.033), lower inspiratory drive (*p* = 0.014), a greater variability of the inter-breath interval (*p* = 0.001) and no significant differences in respiratory (*p* = 0.157) (Table 2).

**Table 2:** Respiratory and additional physiological parameters of infants with and without chorioamnionitis and funisitis

	Infants with HCA + FUN (*n* = 75)	Infants without HCA + FUN (*n* = 111)	Adjusted *p*-value
**Respiratory parameters in the first 5 min after birth**			
Time until start respiratory support (min)	0:54 ± 0:07^a^	0:56 ± 0:07^a^	0.706^a^
Duration of iPPV the first 5 min after birth (min)	1:33 ± 0:14^a^	1:34 ± 0:13^a^	0.965^a^
Inspiratory tidal volume (mL/kg/breath)	3.34 ± 0.45^a^	4.52 ± 0.55^a^	0.003^a^
Respiratory rate (breaths/min)	21 ± 2^a^	19 ± 2^a^	0.205^a^
Incidence of apnoea (n)	4 ± 0^a^	4 ± 0^a^	0.862^a^
Duration per apnoea (s)	25 ± 3^a^	22 ± 3^a^	0.322^a^
Total duration of apnoea (s)	86 ± 12^a^	84 ± 11^a^	0.764^a^
Inter-breath interval COV (%)	81 ± 4^a^	73 ± 4^a^	0.024^a^
Inspiratory drive (mL/kg/breath/s)	7.52 ± 1.00^a^	10.96 ± 1.32^a^	< 0.001^a^
CPAP levels (cm H_2_O)	6.6 ± 0.3^a^	6.9 ± 0.3^a^	0.064^a^
PIP levels (cm H_2_O)	26 ± 1^a^	25 ± 1^a^	0.194^a^
**Additional physiological parameters in the first 5 min after birth**			
HR (beats/min)	122 ± 6^a^	124 ± 6^a^	0.703^a^
Time until HR > 100 bpm (min)	2:18 (1:41–3:33)^b^	2:11 (1:36–3:12)^b^	0.384^b^
SpO_2_ (%)	53 ± 3^a^	57 ± 3^a^	0.067^a^
Time until SpO_2_ > 80% (min)	4:13 (3:18–6:11)^b^	3:52 (2:56–4:47)^b^	0.208^b^
SpO_2_ at 5 min after birth (%)	77 ± 5^a^	85 ± 5^a^	0.021^a^
FiO_2_ (%)	53 ± 3^a^	47 ± 2^a^	0.009^a^
SpO_2_/FiO_2_ ratio (%)	1.15 ± 0.11^a^	1.34 ± 0.12^a^	0.028^a^
Time until stabilisation (min)	8:41 (5:38-NR)^b^	7:05 (5:22–13:56)^b^	0.222^b^
	Infants with subclinical HCA + FUN (*n* = 46)	Infants without any chorioamnionitis (*n* = 102)	
**Respiratory parameters in the first 5 min after birth**			
Time until start respiratory support (min)	1:16 ± 0:08^c^	1:17 ± 0:05^c^	0.964^c^
Duration of iPPV the first 5 min after birth (min)	1:08 ± 0:10^c^	1:09 ± 0:06^c^	0.974^c^
Inspiratory tidal volume (mL/kg/breath)	3.33 ± 0.31^c^	4.41 ± 0.26^c^	0.014^c^
Respiratory rate (breaths/min)	26 ± 2^c^	23 ± 1^c^	0.157^c^
Incidence of apnoea (n)	3 ± 0^c^	3 ± 0^c^	0.791^c^
Duration per apnoea (s)	76 ± 8^c^	77 ± 5^c^	0.964^c^
Total duration of apnoea (s)	86 ± 12^c^	84 ± 11^c^	0.764^c^
Inter-breath interval COV (%)	77 ± 3^c^	67 ± 2^c^	0.009^c^
Inspiratory drive (mL/kg/breath/s)	7.57 ± 0.73^c^	11.18 ± 0.70^c^	0.001^c^
CPAP levels (cm H_2_O)	6.0 ± 0.2^c^	6.4 ± 0.1^c^	0.091^c^
PIP levels (cm H_2_O)	25 ± 0^c^	25 ± 0^c^	0.089^c^
**Additional physiological parameters in the first 5 min after birth**			
HR (beats/min)	121 ± 4^c^	120 ± 3^c^	0.790^c^
Time until HR > 100 bpm (min)	2:11 (1:40–3:28)^d^	2:14 (1:41–3:18)^d^	0.705^d^
SpO_2_ (%)	59 ± 2^c^	63 ± 2^c^	0.168^c^
Time until SpO_2_ > 80% (min)	4:14 (3:16–5:09)^d^	3:59 (2:58–4:52)^d^	0.450^d^
SpO_2_ at 5 min after birth (%)	78 ± 3^c^	85 ± 2^c^	0.036^c^
FiO_2_ (%)	52 ± 2^c^	46 ± 1^c^	0.026^c^
SpO_2_/FiO_2_ ratio (%)	1.27 ± 0.08^c^	1.48 ± 0.07^c^	0.040^c^
Time until stabilisation (min)	7:55 (6:26-NR)^d^	7:18 (6:16–14:55)^d^	0.464^d^

^a^Generalised linear model adjusted for gestational age, antenatal corticosteroids and general anaesthesia

^b^Cox regression adjusted for gestational age, antenatal corticosteroids, and general anaesthesia

^c^Generalised linear model adjusted for gestational age and mode of delivery

^d^Cox regression adjusted for gestational age, antenatal corticosteroids, and general anaesthesia

NR refers to more than 25% of infants not reaching the parameters necessary for stabilisation

Minute volume and tidal volume missing for 1//75 (1%) infants

SpO_2_ data missing for 7/75 (9%), 19/111 (17%), 5/46 (11%) and 18/102 (18%) infants

HR data missing for 7/75 (9%), 20/111 (18%), 5/46 (11%) and 19/102 (19%) infants

FiO_2_ data missing for 3/75 (4%), 4/111 (4%), 3/46 (7%) and 4/102 (4%) infants

### Post-hoc analyses

#### Comparison 1

In post-hoc analyses, HCA + FUN was solely associated with a significant change in respiratory rate over time (*p* = 0.034) (Supplementary File 3).

#### Comparison 2

Subclinical HCA + FUN was associated with a non-significant change in tidal volume (*p* = 0.053) and respiratory rate (*p* = 0.069) over time (Supplementary File 3).

### Additional physiological parameters in the first 5 min after birth

#### Comparison 1

After adjustment for potential confounders, HCA + FUN was associated with lower SpO_2_ levels at 5 min, lower SpO_2_/FiO_2_ ratio and higher FiO_2_ requirements (*p* = 0.009) (Table 2).

#### Comparison 2

Likewise, subclinical HCA + FUN was associated with lower SpO_2_ at 5 min (*p* = 0.036), lower SpO_2_/FiO_2_ ratio (*p* = 0.040) and higher FiO_2_ requirements after adjustment (*p* = 0.026) (Table 2).

### Respiratory support in the delivery room and NICU

#### Comparison 1

After adjustment, HCA + FUN was not associated with any delivery room outcomes, but HCA + FUN was significantly associated with a decreased incidence of surfactant administration in the NICU (0.28 (0.11–0.62) and intubation in the NICU (0.34 (0.14–0.80)) (Table 3).

#### Comparison 2

Similarly, subclinical HCA + FUN was not associated with any delivery room outcomes and was negatively associated with the incidence of surfactant administration in the NICU (0.27 (0.10–0.71)) (Table 3).

**Table 3:** Respiratory support in the delivery room and NICU provided to infants with and without chorioamnionitis and funisitis

	HCA + FUN Adjusted odds ratio (95% CI)	Adjusted *p*-value
**Respiratory support in the delivery room and NICU**		
Initial inflations in the delivery room	1.35 (0.60–3.11)^a^	0.471^a^
iPPV in the delivery room	1.47 (0.69–3.12)^a^	0.314^a^
Intubation in the delivery room	0.93 (0.35–2.41)^a^	0.874^a^
Caffeine administration in the delivery room	0.69 (0.34–1.36)^a^	0.287^a^
Surfactant administration in the NICU	0.28 (0.11–0.62)^a^	0.003^a^
Intubation in the NICU	0.34 (0.14–0.80)^a^	0.038^a^
	Subclinical HCA + FUN Adjusted odds ratio (95%-CI)	
Initial inflations in the delivery room	1.11 (0.45–1.73)^b^	0.815^b^
iPPV in the delivery room	1.45 (0.63–3.41)^b^	0.386^b^
Intubation in the delivery room	1.34 (0.39–4.46)^b^	0.635^b^
Caffeine administration in the delivery room	0.60 (0.25–1.36)^b^	0.224^b^
Surfactant administration in the NICU	0.27 (0.10–0.71)^b^	0.011^b^
Intubation in the NICU	0.45 (0.16–1.15)^b^	0.105^b^

^a^Binary logistic regression adjusted for gestational age, antenatal corticosteroids, mode of delivery and general anaesthesia

^b^Binary logistic regression adjusted for gestational age and mode of delivery

There was no missing data in the table

## Discussion

In this retrospective cohort study, we observed that after adjustment for possible confounders, histological and subclinical chorioamnionitis and funisitis were associated with lower breathing effort parameters, breathing patterns that were more unstable and reduced oxygenation.

We have provided a detailed description of the influence that histological chorioamnionitis and/or funisitis (HCA + FUN) and subclinical HCA + FUN (without clinical chorioamnionitis) could have on breathing effort in premature infants at birth. Our findings are consistent with previous studies that reported less spontaneous breathing and a greater need for oxygen supplementation in premature and very low birth weight infants affected by HCA + FUN [12, 13].

These data suggest that, at birth, the breathing efforts and oxygenation levels of premature infants affected by clinical chorioamnionitis (CCA) might be more adversely affected than those affected by HCA + FUN. In a case–control study, we recently described that CCA was associated with a higher incidence of apnoea, an association we could not confirm for HCA + FUN and subclinical HCA + FUN [16]. However, in the current cohort, HCA + FUN and subclinical HCA + FUN were associated with less stable breathing patterns (higher inter-breath interval COV), indicative of respiratory depression at a lesser extent compared to apnoea. Furthermore, in the current study cohort, the presence of CCA led to marked lower mean breathing effort parameters in both infants affected and unaffected by HCA + FUN. The decrease seemed more pronounced in infants affected by HCA since differences in breathing effort between infants affected by HCA + FUN and infants affected by subclinical HCA + FUN were larger than differences between infants unaffected by HCA + FUN and infants unaffected by any type of chorioamnionitis. In addition, median SpO_2_ levels at 5 min after birth were 9% lower in infants affected by CCA (case–control study) compared to infants affected by HCA + FUN (current cohort) and 13% lower in infants affected by CCA (case–control study) compared to infants affected by subclinical HCA + FUN (current cohort) [16]. Experimental studies have shown that both prostaglandins and adenosine can suppress breathing in a dose-dependent way, and cause apnoea at higher concentrations while lowering tidal volume at lower concentrations [23, 24]. It is possible that infants affected by CCA were exposed to higher concentrations of these mediators, leading to a suppression of breathing, lower oxygen saturation (SpO_2_) levels and higher incidence of apnoea. This is also consistent with our finding of initial lower tidal volume and respiratory rate values in infants affected by HCA + FUN compared to infants affected by subclinical HCA + FUN as well as sustained lower tidal volume in infants affected by HCA + FUN, which was less evident in infants affected by subclinical chorioamnionitis (Supplemental file 4). This may be because the latter are exposed to lower concentrations of inflammatory and hypoxic mediators.

While we found that subclinical HCA + FUN was associated with lower SpO_2_ levels 5 min after birth, a previous study found no evidence for such a difference [15]. However, they investigated term infants whereas we only included premature infants < 30 weeks’ gestation, so the association between subclinical HCA + FUN and reduced oxygenation might not hold true for term infants [15]. Nevertheless, even with a similar SpO_2_, they observed higher intubation rates at birth in term infants with subclinical HCA + FUN; a finding we were not able to reproduce [15].

In infants affected by HCA + FUN and subclinical HCA + FUN, a lower breathing effort that failed to increase over time, combined with more a unstable breathing pattern, might have delayed lung aeration and contributed to reduced oxygenation in these infants. This lower breathing effort was largely due to lower tidal volumes, particularly less depth of breathing, which possibly reflects a centrally mediated lowered inspiratory drive. Alternatively, it could also be a consequence of reduced lung compliance associated with either incomplete lung liquid clearance or increased lung tissue inflammation as well as decreased diaphragmatic contractility [25–27]. All of these factors have been observed in experimental studies to lower inspiratory effort [25–27]. Moreover, the interplay of chemical inhibition on respiratory drive, physical obstruction, changes in pulmonary function and a more unstable breathing pattern that reduces laryngeal patency, could all delay lung aeration [2]. Delayed lung aeration limits the surface area available for gas exchange due to the presence of lung liquid within the gas exchange region of the lung [1]. By reducing the surface area, the capacity for oxygen diffusion across the alveolar-capillary barrier is reduced [28]. Thus, the combined effect of reduced respiratory drive and a reduced gas exchange surface area may explain why infants affected by HCA + FUN and subclinical HCA + FUN were associated with lower SpO_2_ levels at 5 min despite significantly higher inspired oxygen levels [25].

This study is limited by its retrospective nature. In particular, missing data likely provided a bias, as the baseline characteristics of infants from pregnancies with available placental pathology differed significantly from those without (Supplemental file 2). This bias also limits the generalisability of the study, because placental pathology was mostly determined in pregnancies with complications; infants were younger, lighter, more often exposed to general anaesthesia and had lower Apgar scores. However, this bias is more likely to underestimate the effect sizes, as we have not compared premature infants affected by (subclinical) HCA + FUN with a normal control group of infants, but are compared with premature infants complicated by other conditions. Thus, our findings, which likely underestimate the effect size, are still generalisable to premature infants affected by (subclinical) HCA + FUN but have to be taken into appropriate consideration. Moreover, notwithstanding a lack of specific criteria for defining, grading and differentiating chorioamnionitis and funisitis, we collected data on inflammatory location known to affect premature infants at birth and our incidence of HCA + FUN was within range of the literature [29], therefore allowing our findings to be reproducible for others.

As antenatal inflammation, in any form (CCA, HCA + FUN or subclinical HCA + FUN) affects a large proportion of premature infants, clinicians should be aware of the possible negative association between antenatal inflammation and spontaneous breathing [4, 8, 16]. As HCA + FUN often occurs subclinical, stimulating and supporting spontaneous breathing of all premature infants is essential because caregivers do not know what infants are at risk for depressed breathing. Furthermore, our findings create a rationale for further research into how the effect of antenatal inflammation on breathing effort could be reduced or prevented, possibly via therapeutic agents aimed at lowering inflammation, hypoxia or antimicrobial load [1].

## Conclusion

Histological and subclinical chorioamnionitis and funisitis are both associated with reduced breathing effort parameters and oxygenation levels in premature infants at birth, which might indicate that in the presence of antenatal inflammation, premature infants may be exposed to higher concentrations of inhibitors that suppress respiratory drive.

## Supplementary Information

Below is the link to the electronic supplementary material.

Supplementary file1 (DOCX 14 KB)

Supplementary file2 (DOCX 16 KB)

Supplementary file3 (DOCX 23 KB)

Supplementary file4 (DOCX 205 KB)

## Data Availability

The datasets generated during and/or analysed during the current study are not publicly available due to patient privacy.

## Abbreviations

COV: Coefficient of variation
CCA: Clinical chorioamnionitis
CPAP: Continuous positive airway pressure
FiO_2_: Fraction of inspired oxygen
FUN: Funisitis
HCA: Histological chorioamnionitis
HCA + FUN: Histological chorioamnionitis and/or funisitis
HR: Heart rate
LUMC: Leiden University Medical Centre
MV: Minute volume
NICU: Neonatal Intensive Care Unit
NR: Not reached
iPPV: Intermittent positive pressure ventilation
PIP: Peak inspiratory pressure
RFM: Respiratory function monitor
RR: Respiratory rate
SpO_2_: Oxygen saturation
Vt: Tidal volume

## Footnotes

1 Maternal intrapartum antibiotic use comprised the following antimicrobials: amoxicillin, amoxicillin-clavulanate, cefuroxime, benzylpenicillin, gentamicin, metronidazole and clindamycin.

2 CCA was diagnosed based on the suspected Triple I criteria, comprising fever of the pregnant woman with one of the following symptoms: leukocytosis, fetal tachycardia or purulent vaginal discharge 9.Peng CC, Chang JH, Lin HY, Cheng PJ, Su BH. Intrauterine inflammation, infection, or both (Triple I): A new concept for chorioamnionitis. Pediatr Neonatol. 2018;59(3):231–7.

3 Time until a HR/SpO_2_/FiO_2_ was reached.
