# Supplementary material for: The effect of histological and subclinical chorioamnionitis and funisitis on breathing effort in premature infants at birth: a retrospective cohort study
Source: Eur J Pediatr. 2024 Oct 25;183(12):5497–507. doi: 10.1007/s00431-024-05815-w (PMC11527944; doi:10.1007/s00431-024-05815-w)
Supplement: Supplementary file 1 — Supplementary file1 (DOCX 14 KB) [file 431_2024_5815_MOESM1_ESM.docx]

Indications for placental pathology:

- intrauterine death without a known cause;
- perinatal asphyxia (infant with Apgar score ≤5 at five minutes and pH ≤7.0);
- unexplained fetal growth restriction with a weight below the 2.3 percentile;
- doubtful (partial) abruption;
- intrauterine infection with an unknown pathogen;
- unexplained preterm birth at <34 weeks gestation;
- discordant growth in dichorionic twins (based on birth weight discordance >20%);
- monochorionic twin pregnancy;
- rhesus immunization with and without intrauterine transfusions;
- partial molar pregnancy or placental tumours;
- macroscopically abnormal placenta;
- MIRROR syndrome (fetal and placental hydrops preeclampsia), and;
- multiple congenital anomalies without prenatal diagnosis and in cases of rhesus immunization with and without intrauterine transfusions
