# Supplementary material for: The effect of histological and subclinical chorioamnionitis and funisitis on breathing effort in premature infants at birth: a retrospective cohort study
Source: Eur J Pediatr. 2024 Oct 25;183(12):5497–507. doi: 10.1007/s00431-024-05815-w (PMC11527944; doi:10.1007/s00431-024-05815-w)
Supplement: Supplementary file 2 — Supplementary file2 (DOCX 16 KB) [file 431_2024_5815_MOESM2_ESM.docx]

Table 0: Baseline characteristics of infants born from pregnancies with and without placenta pathology reports

|  | **Infants born from pregnancies with available placenta pathology report (n=186)** | **Infants born from pregnancies without available placenta pathology report (n=135)** | P-value |
| --- | --- | --- | --- |
|  |  |  |  |
| **Baseline characteristics** |  |  |  |
|  |  |  |  |
| Maternal age (yrs) | 31±5 | 30±5 | 0.634^a^ |
| Primiparity | 112 (60%) | 84 (62%) | 0.716^b^ |
| Multiple gestation | 55 (30%) | 81 (60%) | <0.001^b^ |
| Caesarean section | 98 (53%) | 77 (57%) | 0.600^b^ |
| General anaesthesia | 38 (20%) | 16 (12%) | 0.043^b^ |
| Full course of antenatal corticosteroids | 119 (64%) | 101 (75%) | 0.111^b^ |
| Maternal intrapartum antibiotics | 54 (29%) | 48 (36%) | 0.215^b^ |
| Clinical chorioamnionitis | 38 (20%) | 15 (11%) | 0.026^b^ |
| Gestational age (weeks) | 27^+5^ (25^+6^-29^+0^) | 28^+1^ (27^+0^-29^+0^) | <0.001^c^ |
| Birthweight (g) | 991±270 | 1075±255 | 0.003^b^ |
| Small for gestational age | 86 (46%) | 76 (56%) | 0.263^b^ |
| Male | 86 (46%) | 76 (56%) | 0.075^b^ |
| Apgar score 1 min | 5 (2-7) | 6 (3-7) | 0.006^c^ |
| Apgar score 5 min | 7 (6-8) | 8 (7-9) | 0.097^c^ |
| Umbilical pH | 7.27±0.14 | 7.26±0.10 | 0.528^a^ |
|  |  |  |  |
| Umbilical pH represents the umbilical artery or vein pH Small for gestational age is defined as an infants with birthweight <p10 for gestational age.  ^a^ Independent Samples T-test ^b^ Chi^2^-test ^c^ Mann-Whitney U test Umbilical pH data missing for 49/186 (26%) and 38/135 (28%) infants. | | | |
