# Supplementary material for: The effect of histological and subclinical chorioamnionitis and funisitis on breathing effort in premature infants at birth: a retrospective cohort study
Source: Eur J Pediatr. 2024 Oct 25;183(12):5497–507. doi: 10.1007/s00431-024-05815-w (PMC11527944; doi:10.1007/s00431-024-05815-w)
Supplement: Supplementary file 3 — Supplementary file3 (DOCX 23 KB) [file 431_2024_5815_MOESM3_ESM.docx]

Raw data: Respiratory and additional physiological parameters of infants with and without chorioamnionitis and funisitis

|  | **Infants with HCA+FUN  (n=75)** | **Infants without HCA+FUN (n=111)** | P-value |
| --- | --- | --- | --- |
|  |  |  |  |
| **Respiratory parameters in the first 5 minutes after birth** | | | |
|  |  |  |  |
| Time until start respiratory support (min) | 0:56 (0:34-1:18) | 1:09 (0:44-1:49) | 0.016^a^ |
| Duration of iPPV the first 5 minutes after birth (min) | 1:17 (0:00-2:23) | 0:43 (0:00-2:04) | 0.032^a^ |
| Inspiratory tidal volume (mL/kg/breath) | 3.13±1.72 | 4.66±3.07 | <0.001^b^ |
| Respiratory rate (breaths/min) | 18 (10-28) | 25 (14-32) | 0.043^a^ |
| Incidence of apnoea (n) | 3 (1-5) | 2 (0-4) | 0.057^a^ |
| Duration per apnoea (s) | 20 (15-26) | 17 (14-21) | 0.033^a^ |
| Total duration of apnoea (s) | 70 (18-122) | 35 (0-86) | 0.016^a^ |
| Inter-breath interval COV (%) | 73±22 | 69±19 | 0.182^b^ |
| Inspiratory drive (mL/kg/breath/s) | 6.58 (4.79-9.40) | 10.83 (6.37-15.36) | <0.001^a^ |
| CPAP levels (cm H_2_O) | 6±1 | 6±1 | 0.101^b^ |
| PIP levels (cm H_2_O) | 25±2 | 24±3 | 0.023^b^ |
|  |  |  |  |
| **Additional physiological parameters in the first 5 minutes after birth** | | | |
|  |  |  |  |
| HR (beats/min) | 115±26 | 120±26 | 0.240^b^ |
| Time until HR >100 bpm (min) | 2:18 (1:44-3:32) | 2:10 (1:30-3:19) | 0.422^c^ |
| SpO_2_ (%) | 59±14 | 63±14 | 0.063^b^ |
| Time until SpO_2_ >80% (min) | 4:03 (3:24-5:30) | 3:49 (2:51-4:45) | 0.111^c^ |
| SpO_2_ at 5 minutes after birth (%) | 86 (65-93) | 93 (82-99) | 0.025^a^ |
| FiO_2_ (%) | 54 (39-64) | 45 (34-55) | 0.001^a^ |
| SpO_2_/FiO_2_ ratio (%) | 1.18±0.51 | 1.51±0.62 | <0.001^b^ |
| Time until stabilisation (min) | 9:38 (6:00-NR) | 6:52 (5:16-12:12) | 0.054^c^ |
|  |  |  |  |
|  | **Infants with subclinical HCA+FUN (n=46)** | **Infants without subclinical HCA+FUN (n=102)** |  |
|  |  |  |  |
| **Respiratory parameters in the first 5 minutes after birth** | | | |
|  |  |  |  |
| Time until start respiratory support (min) | 0:55 (0:30-1:27) | 1:12 (0:44-1:51) | 0.039^a^ |
| Duration of iPPV the first 5 minutes after birth (min) | 1:13 (0:00-2:30) | 0:29 (0:00-1:52) | 0.048^a^ |
| Inspiratory tidal volume (mL/kg/breath) | 3.19±1.82 | 4.76±3.12 | <0.001^b^ |
| Respiratory rate (breaths/min) | 19 (11-30) | 25 (15-33) | 0.104^a^ |
| Incidence of apnoea (n) | 4 (1-5) | 2 (0-4) | 0.049^a^ |
| Duration per apnoea (s) | 18 (15-25) | 17 (14-20) | 0.072^a^ |
| Total duration of apnoea (s) | 69 (13-112) | 30 (0-81) | 0.038^a^ |
| Inter-breath interval COV (%) | 75±22 | 68±19 | 0.031^b^ |
| Inspiratory drive (mL/kg/breath/s) | 6.47 (4.56-9.76) | 10.83 (6.50-15.51) | <0.001^a^ |
| CPAP levels (cm H_2_O) | 6±1 | 6±1 | 0.094^b^ |
| PIP levels (cm H_2_O) | 26±2 | 24±3 | 0.007^b^ |
|  |  |  |  |
| **Additional physiological parameters in the first 5 minutes after birth** | | | |
|  |  |  |  |
| HR (beats/min) | 119±26 | 121±26 | 0.701^b^ |
| Time until HR >100 bpm (min) | 2:14 (1:41-3:20) | 2:12 (1:41-2:59) | 0.802^c^ |
| SpO_2_ (%) | 59±16 | 64±13 | 0.114^b^ |
| Time until SpO_2_ >80% (min) | 4:10 (3:25-5:12) | 3:49 (2:51-4:49) | 0.276^c^ |
| SpO_2_ at 5 minutes after birth (%) | 90 (69-93) | 90 (80-95) | 0.154^a^ |
| FiO_2_ (%) | 57 (40-67) | 44 (34-54) | <0.001^a^ |
| SpO_2_/FiO_2_ ratio (%) | 1.15±0.53 | 1.55±0.62 | 0.001^b^ |
| Time until stabilisation (min) | 10:16 (6:00-NR) | 6:51 (5:06-12:15) | 0.064^c^ |
|  |  |  |  |
| ^a^ Mann-Whitney U test ^b^ Independent Samples-T test  ^c^ Log-rank Test  NR refers to more than 25% of infants not reaching parameters necessary for stabilisation.  Minute volume and tidal volume missing data for 1/75 (1%) of infants.  SpO_2_ data missing for 7/75 (9%), 19/111 (17%), 5/46 (11%) and 18/102 (18%) infants HR data missing for 7/75 (9%), 20/111 (18%), 5/46 (11%) and 19/102 (19%) infants FiO_2_ data missing for 3/75 (4%), 4/111 (4%), 3/46 (7%) and 4/102 (4%) infants | | | |

Raw data: Respiratory support in the delivery room and NICU provided to infants with and without chorioamnionitis and funisitis

|  | **Infants with HCA+FUN  (n=75)** | **Infants without HCA+FUN (n=111)** | P-value |
| --- | --- | --- | --- |
|  |  |  |  |
| **Respiratory support in the delivery room and NICU** | | | |
|  |  |  |  |
| Initial inflations in the delivery room | 62 (83%) | 80 (72%) | 0.095^a^ |
| iPPV in the delivery room | 54 (72%) | 61 (55%) | 0.019^a^ |
| Intubation in the delivery room | 16 (21%) | 13 (12%) | 0.076^a^ |
| Caffeine administration in the delivery room | 40 (54%) | 57 (51%) | 0.718^a^ |
| Surfactant administration in the NICU | 44 (58.7%) | 67 (60%) | 0.817^a^ |
| Intubation in the NICU | 46 (61%) | 59 (53%) | 0.270^a^ |
|  |  |  |  |
|  | **Infants with subclinical HCA+FUN (n=46)** | **Infants without subclinical HCA+FUN (n=102)** |  |
|  |  |  |  |
| Initial inflations in the delivery room | 35 (76%) | 72 (71%) | 0.489^a^ |
| iPPV in the delivery room | 31 (67%) | 52 (51%) | 0.063^a^ |
| Intubation in the delivery room | 8 (17%) | 9 (9%) | 0.130^a^ |
| Caffeine administration in the delivery room | 22 (48%) | 52 (51%) | 0.722^a^ |
| Surfactant administration in the NICU | 23 (50%) | 59 (58%) | 0.374^a^ |
| Intubation in the NICU | 27 (53%) | 52 (51%) | 0.384^a^ |
|  |  |  |  |
| ^a^ Chi^2^-test | | | |
