# Supplementary material for: The effect of histological and subclinical chorioamnionitis and funisitis on breathing effort in premature infants at birth: a retrospective cohort study
Source: Eur J Pediatr. 2024 Oct 25;183(12):5497–507. doi: 10.1007/s00431-024-05815-w (PMC11527944; doi:10.1007/s00431-024-05815-w)
Supplement: Supplementary file 4 — Supplementary file4 (DOCX 205 KB) [file 431_2024_5815_MOESM4_ESM.docx]

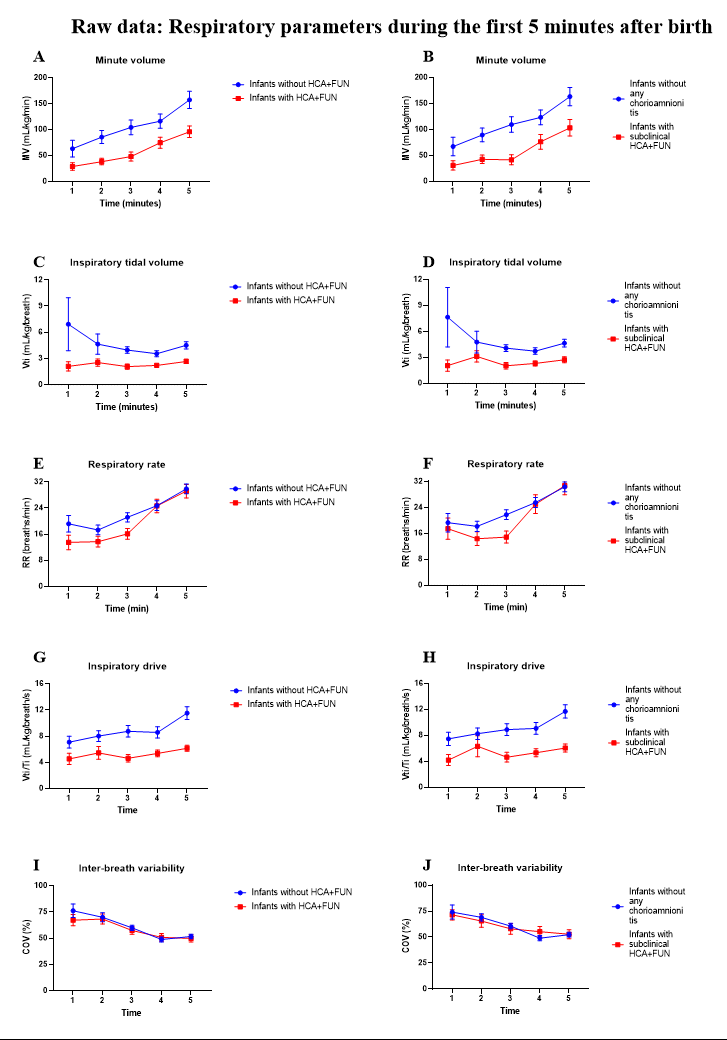


Raw measurements of minute volume **(A, B)**, tidal volume **(C, D)**, respiratory rate **(E, F)**, inspiratory drive **(G, H)**, and inter-breath variability **(I, J)** with mean and standard error in premature infants with and without HCA+FUN (left side) and infants with subclinical HCA+FUN and infants without any chorioamnionitis (right side) during the first 5 minutes after birth.


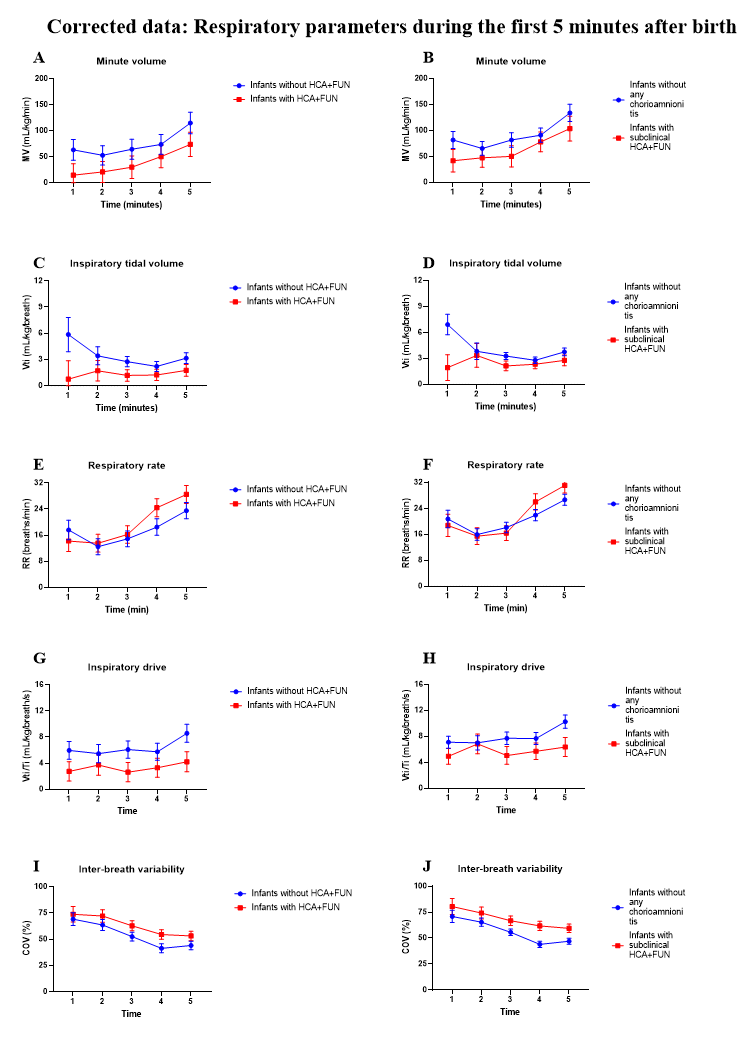


Corrected measurements of minute volume **(A, B)**, tidal volume **(C, D)**, respiratory rate **(E, F)**, inspiratory drive **(G, H)**, and inter-breath variability **(I, J)** with mean and standard error in premature infants with and without HCA+FUN (left side) and infants with subclinical HCA+FUN and infants without any chorioamnionitis (right side) during the first 5 minutes after birth.
